# AZD3759 inhibits glioma through the blockade of the epidermal growth factor receptor and Janus kinase pathways

**DOI:** 10.1080/21655979.2021.1991160

**Published:** 2021-10-25

**Authors:** Wei Yin, Ke Zhang, Qinghua Deng, Qingqing Yu, Yanjiao Mao, Ruping Zhao, Shenglin Ma

**Affiliations:** a Department of Radiation Oncology, Hangzhou Cancer Hospital, Hangzhou, Zhejiang, China; b Department of Radiation Oncology, Jiahui International Hospital, Shanghai, China

**Keywords:** AZD3759, glioma, osimertinib, epidermal growth factor receptor, Janus kinase

## Abstract

Glioma is an intracranial malignant tumor with high morbidity in China. Limited efficacy has been achieved in the treatment of glioma through the application of epidermal growth factor receptor (EGFR) inhibitors, which is reported to be related to the poor permeability of the brain–blood barrier (BBB) to EGFR inhibitors. AZD3759 and osimertinib are both BBB-penetrating EGFR inhibitors. The present study aimed to investigate the inhibitory effects of AZD3759 and osimertinib on glioma and compare their efficacy and the underlying mechanisms. C6 and U87 cells were incubated with different concentrations of AZD3759 (1, 2, and 4 μM) and 4 μM osimertinib, respectively. C6-LUC xenograft animals were administered different doses of AZD3759 (15, 30, and 60 mg/kg) and 60 mg/kg osimertinib. We found that proliferation was significantly suppressed and that apoptosis and cell cycle arrest were dramatically induced in both C6 and U87 cells by AZD3759 in a dose-dependent manner. Compared to AZD3759, osimertinib had inferior effects on proliferation, apoptosis, and cell cycle. *In vivo* experiments verified that the anti-tumor efficacy of AZD3759 against C6 xenograft tumors was dose dependent and superior to that of osimertinib. The inhibitory effects of AZD3759 on the Janus kinase (JAK)/STAT pathway were observed in both glioma cells and tumor tissues, which were more significant than those of osimertinib. In conclusion, AZD3759 may inhibit the progression of glioma via a synergistic blockade of the EGFR and JAK/STAT signaling pathways.

## Introduction

Glioma is a type of malignant tumor commonly observed in the Department of Neurosurgery, and the average annual morbidity of glioma in China is approximately 0.003%–0.008%, which is mainly induced by the synergic actions of congenital genetic risk factors and environmental carcinogenic factors [1]. In the clinic, the main characteristics of glioma include seizures, headaches, and blurred vision, which significantly affect the normal life of patients with glioma [2]. Currently, excision of tumors to a maximum extent with chemotherapy is the standard therapy for the treatment of clinical glioma. However, due to its invasive and infiltrative growth, the total resection rate is low and significant resistance to radiotherapy and chemotherapy is observed in the remaining tumor tissues [3]. Therefore, the development of blood–brain barrier (BBB)-penetrative targeted agents for the treatment of glioma is urgently needed.

Owing to the development of molecular oncology, novel targeted therapies for the treatment of glioma have been developed, among which the epidermal growth factor receptor (EGFR) is an important target. The EGFR is a transmembrane receptor tyrosine kinase (RTK) that is critical for normal cellular function and is significantly upregulated in malignant tumor cells [4,5]. EGFRs are highly expressed in tumor tissues of patients with glioma, and 20%–30% patients carry the EGFR VIII mutant gene, which is the mutant tumor gene commonly observed in glioma [6,7]. Persistent activation of RTK can be induced by EGFR VIII mutations [8]. Although multiple new drugs targeting EGFR have been approved for phase I and II clinical trials, higher efficacies than that of temozolomide have been rarely reported [9]. The main factors responsible for the low efficacy include the heterogeneity of EGFR molecules and glioma tissues [10], complication of EGFR downstream signaling pathways [11], and difficulty of EGFR antagonists to pass through the BBB [12]. Therefore, molecules with high selectivity to EGFR mutant genes and high penetration through the BBB may be effective for the treatment of glioma.

AZD3759, a reformatted EGFR-TKI of gefitinib, has been shown to pass through the BBB to enter the central nervous system [13]. The penetrability of AZD3759 through the BBB is higher than that of erbtinib and gefitinib. In a previous study, the growth of intracranial tumors in a brain metastasis mouse model was significantly inhibited by treatment with AZD3759, which was accompanied by a prolonged survival in mice [14]. In addition, clinical trials have shown higher concentrations of AZD3759 in the central nervous system and its promising anti-tumor efficacy [15]. We hypothesized that AZD3759 inhibits the development of glioma by repressing the EGFR and JAK pathways. In the present study, the anti-tumor efficacy of AZD3759 against glioma and its underlying mechanism was investigated to provide more clinical evidence for the application of AZD3759 for the treatment of patients with glioma and EGFR-sensitive mutations in the clinic.

## Materials and methods

### Cells and agents

The C6 rat glioma cell line, U87 human glioma cell line, and luciferase labeled C6 cells (C6-LUC) were purchased from ATCC (Maryland, USA). They were cultured in Dulbecco’s modified Eagle medium supplemented with 10% fetal bovine serum at 5% CO_2_ and 37°C. Osimertinib and AZD3759 were purchased from AbMole (Texas, USA). Osimertinib and AZD3759 stock solutions were prepared in dimethyl sulfoxide, which was diluted in serum-free culture medium before being introduced in *in vitro* assays. For *in vivo* experiments, a vehicle (0.5% methylcellulose [w/v] and 0.4% Tween 80 [v/v] in sterile water) was used to dissolve osimertinib and AZD3759 for oral administration to animals.

### Clonogenic assay*[16]*

C6 cells or U87 cells were seeded on six-well plates and treated with osimertinib (4 μM) or AZD3759 (1, 2, and 4 μM) for 10–14 consecutive days until macroscopic clones were observed. Cells were stained with 0.5% crystal violet (Sigma, Missouri, USA) in 10% methanol for 30 min, which was followed by counting of colonies with >50 cells. The surviving fraction was calculated at each concentration by dividing the total number of colonies after treatment by the number of colonies without treatment.

### Flow cytometry for the analysis of apoptosis*[17]*

C6 cells or U87 cells were seeded on six-well plates at a density of 2 × 10^5^ cells/well and treated with osimertinib (4 μM) or AZD3759 (1, 2, and 4 μM) for 24 h, followed by resuspension in 500 μL annexin V binding buffer containing 5 μL PI and 5 μL annexin V-FITC. This was followed by flow cytometry (BD, New Jersey, USA) to determine the apoptotic rate in each group.

### Flow cytometry for the analysis of cell cycle*[18]*

C6 cells, U87 cells, or C6-LUC cells were seeded on six-well plates at a density of 2 × 10^5^ cells/well and treated with osimertinib (4 μM) or AZD3759 (1, 2, and 4 μM) for 24 h. This was followed by centrifugation at 750 × g for 5 min and three washes with phosphate-buffered saline buffer. Subsequently, the cells were counted and fixed in 70% ice-cold ethanol overnight at 4°C. After centrifugation at 1200 × g for 5 min, the cells were resuspended and stained with 50 μg/mL PI and 50 μg/mL RNase at 37°C for 30 min. Finally, cell cycle analysis was performed using flow cytometry (BD, New Jersey, USA).

### Western blotting assay

Total protein was obtained from cells or tissues using a lysis buffer (Cell Signaling Technology, California, USA), and protein levels were quantified using the BCA kit (Shanghai Ze Ye Biotechnology Co., Ltd, Shanghai, China). Subsequently, approximately 30 μg of protein was loaded and separated by 12% sodium dodecyl sulfate–polyacrylamide gel electrophoresis. The proteins were then transferred to a polyvinylidene fluoride membrane (Millipore, Massachusetts, USA), followed by incubation with 5% bovine serum albumin and incubation with the primary antibodies against JAK1 (1:800, Invitrogen, Massachusetts, USA), p-JAK1 (1:800, Invitrogen, Massachusetts, USA), JAK2 (1:800, Invitrogen, Massachusetts, USA), p-JAK1 (1:800, Invitrogen, Massachusetts, USA), STAT3 (1:800, Invitrogen, Massachusetts, USA), p-STAT3 (1:800, Invitrogen, Massachusetts, USA), STAT5 (1:800, Invitrogen, Massachusetts, USA), p-STAT5 (1:800, Invitrogen, Massachusetts, USA), and GAPDH (1:800, Invitrogen, Massachusetts, USA). Subsequently, the membranes were incubated with a secondary antibody (1:2000; Invitrogen, Massachusetts, USA). Finally, enhanced chemiluminescence (Invitrogen, Massachusetts, USA) was used for the visualization of bands, followed by quantification using the ImageJ software (National Institutes of Health, USA) [19].

### Detection of luciferin activity in C6-LUC cells*[20]*

C6-LUC cells in the logarithmic phase were seeded on 96-well plates with different cell numbers (4 × 10^4^, 2 × 10^4^, l × 10^4^, 5000, 2500, 1250, 625, 312, and 156). A blank medium was used as a negative control. After incubation for 24 h, the substrate of luciferase was added at a concentration of 150 μg/mL, and the IVIS spectrum system (PerkinElmer, Massachusetts, USA) was used to detect the activity of luciferin immediately.

### CCK-8 assay*[21]*

Briefly, C6-LUC cells were mixed with a 10-µL CCK-8 solution and incubated at 37°C for 2 h. The absorbance at 450 nm was then measured using a microplate reader (J&H technology Co., Ltd, Jiangsu, China) to determine cell viability.

### Establishment of animal models*[20]*

Approximately 7–8-week-old imprinting control region male mice were purchased from Charles River Laboratories (Beijing, China) and adapted in our laboratory for 2 weeks. C6-LUC cells were collected and washed with Hank’s solution. The concentration of C6 cells used for planting was set at 1.0 × 10^6^/15 μL/animal. Mice were intraperitoneally injected with 5% chloral hydrate and fixed on a brain stereotaxic apparatus. The skin on the right forehead was prepared and sterilized with 75% ethanol, and a vertical incision was made on the scalp along the bregma to expose the skull. The skull was then drilled 0.4 mm anterior to the coronal suture and 2 mm to the right of the sagittal suture. The tip of the needle was vertically and slowly inserted 3.0 mm beneath the endocranium along the hole to gradually inject C6-LUC cells into the brain. The needle was removed 1 min after injection, and the hole was closed with bone wax. Lastly, the incision was stitched, the skin was sterilized, and penicillin was injected intramuscularly.

### In vivo *grouping*

When the intracranial fluorescent signal in animals was >5 × 10^6^ photons/s, mice were treated with different strategies. The mice were divided into the following five groups: control (mice implanted with C6-LUC cells that were administered vehicle), 15 mg/kg/day AZD3759 (mice implanted with C6-LUC cells that were administered 15 mg/kg/day AZD3759), 30 mg/kg/day AZD3759 (mice implanted with C6-LUC cells that were administered 30 mg/kg/day AZD3759), 60 mg/kg/day AZD3759 (mice implanted with C6-LUC cells that were administered 60 mg/kg/day AZD3759), and 25 mg/kg/day osimertinib (mice implanted with C6-LUC cells that were administered 25 mg/kg/day osimertinib). The treatment duration for all groups was 3 weeks. Tumor growth was monitored by measuring bioluminescence signals at 1 week and 3 weeks after planting. Tumors were isolated from sacrificed animals at the end of the experiments, and tumor volumes were calculated by measuring the length and width of the tumors.

## Statistical analysis

Data were analyzed using the GraphPad software and are presented as mean ± standard deviation. The t-test was used to compare two independent data, and the data among groups were compared using one-way analysis of variance. A p-value of <0.05 was considered to indicate a significant difference.

## Results

We hypothesized that development of glioma is repressed by AZD3759 through the inhibition of the EGFR and JAK pathways. The present study aimed to investigate the anti-tumor efficacy of AZD3759 against glioma and determine its underlying mechanism to provide more clinical evidence for the application of AZD3759 for the treatment of patients with glioma and EGFR-sensitive mutations in clinical settings. We first evaluated the inhibitory effects of AZD3759 on the proliferation of glioma cells and the evaluated of the regulatory effects of AZD3759 on the EGFR and JAK pathways in glioma cells. Subsequently, C6-LUC cells were established and implanted into nude mice, followed by treatment with AZD3759 and osimertinib. The growth of glioma cells in nude mice was monitored. Lastly, the effects of AZD3759 and osimertinib on the EGFR and JAK pathways in tumor-bearing mice were confirmed.

### AZD3759 suppressed proliferation and induced apoptosis in glioma cells

**Figure 1.**
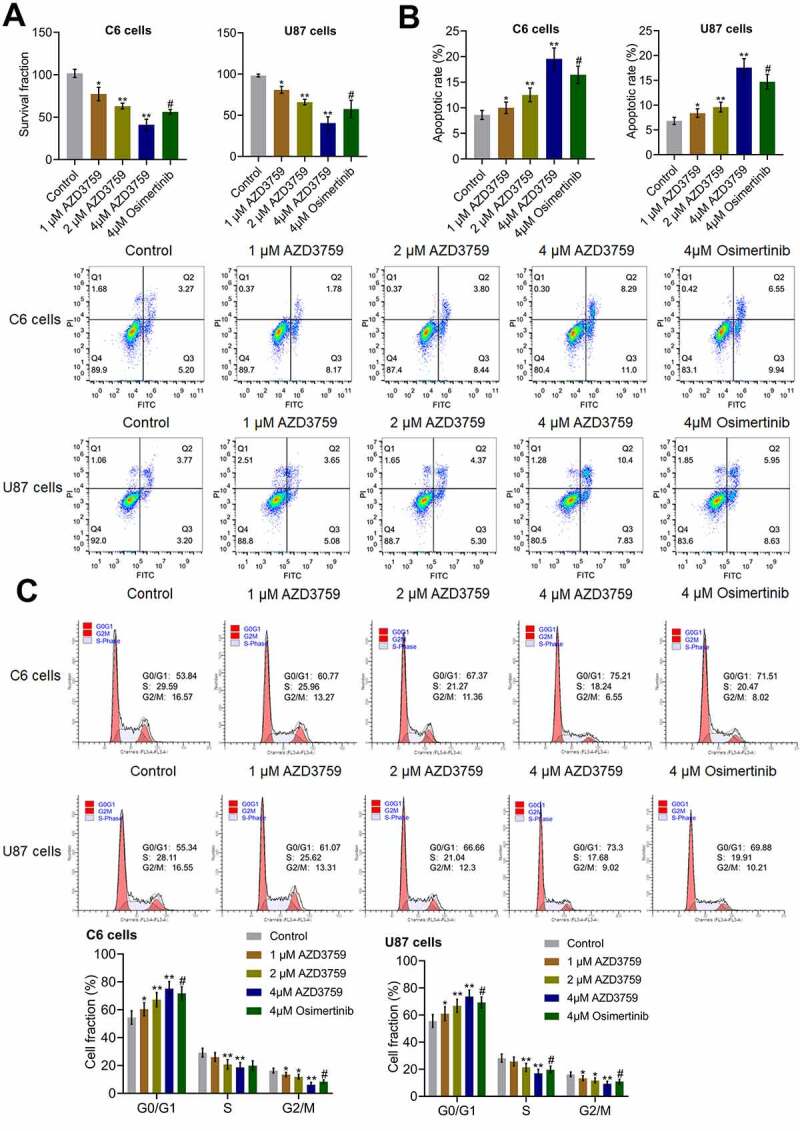
Proliferation is suppressed and apoptosis is induced by AZD3759 in glioma cells. a. The clonogenic assay used to determine the proliferation ability of cells. b. The apoptotic rate of treated glioma cells detected by flow cytometry. The Q2 + Q3 flow cytometry gates are used for the calculation of apoptotic rate. c. The cell cycle of treated glioma cells detected by flow cytometry (*p < 0.05 vs. control, **p < 0.01 vs. control, #p < 0.05 vs. 4 μM AZD3759)

To explore the inhibitory effect of AZD3759 on the growth of glioma cells, the proliferation and apoptosis of both murine and human glioma cells were analyzed after treatment with different concentrations of AZD3759 or 4 μM osimertinib. As shown in Figure 1a, compared to the control treatment, treatment with AZD3759 significantly declined the survival fraction in C6 cells and U87 cells in a dose-dependent manner (*p < 0.05 vs. control, **p < 0.01 vs. control). Compared to the 4 μM AZD3759 group, the 4 μM osimertinib group had a significantly higher survival fraction in the (#p < 0.05 vs. 4 μM AZD3759). In addition, compared to control treatment, treatment with 1 μM, 2 μM, and 4 μM AZD3759 dramatically increased the apoptotic rate (Figure 1b) from 8.47% to 9.95%, 12.24%, and 19.29%, respectively, in C6 cells and 6.97% to 8.73%, 9.67%, and 18.23%, respectively, in U87 cells (*p < 0.05 vs. control, **p < 0.01 vs. control). In both C6 and U87 cells, the apoptotic rate was significantly lower in the 4 μM osimertinib group than in the 4 μM AZD3759 group (#p < 0.05 vs. 4 μM AZD3759).

As shown in Figure 1b, the cell fraction at the G0/G1 phase was significantly elevated from 53.84% to 60.77%, 67.37%, and 75.21% in C6 cells and increased from 55.34% to 61.07%, 66.66%, and 73.3% in U87 cells after incubation with 1 μM, 2 μM, and 4 μM AZD3759, respectively, compared to those after control treatment (*p < 0.05 vs. control, **p < 0.01 vs. control). In both C6 and U87 cells, compared to the 4 μM AZD3759 group, the 4 μM osimertinib group had a significantly lower cell fraction at the G0/G1 phase (#p < 0.05 vs. 4 μM AZD3759).

### AZD3759 inhibited the EGFR and JAK/STAT signaling pathways in glioma cells

**Figure 2.**
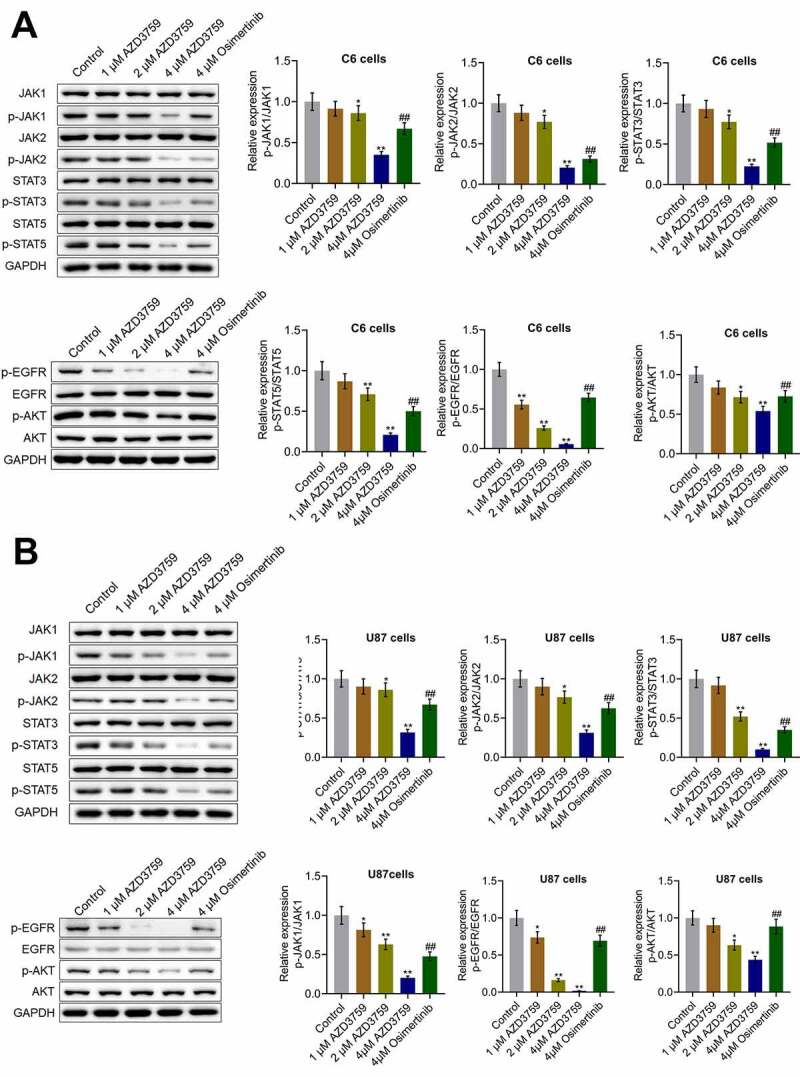
The EGFR and JAK/STAT pathways in glioma cells are inhibited by AZD3759. a. The expression levels of JAK1, p-JAK1, JAK2, p-JAK2, STAT3, p-STAT3, STAT5, p-STAT5, EGFR, p-EGFR, AKT, and p-AKT in C6 cells detected using western blot assay. b. The expression levels of JAK1, p-JAK1, JAK2, p-JAK2, STAT3, p-STAT3, STAT5, p-STAT5, EGFR, p-EGFR, AKT, and p-AKT in U87 cells detected using western blot assay (*p < 0.05 vs. control, **p < 0.01 vs. control, ##p < 0.01 vs. 4 μM AZD3759)

We first examined the effects of AZD3759 on the EGFR pathway. As shown in Figure 2, in both C6 cells and U87 cells, the expression levels of p-EGFR/EGFR and p-AKT/AKT were dramatically repressed by treatment with AZD3759 in a dose-dependent manner compared to those by control treatment (*p < 0.05 vs. control, **p < 0.01 vs. control). Compared to the 4 μM AZD3759 group, the 4 μM osimertinib group had a significantly higher expression of p-EGFR/EGFR and p-AKT/AKT (##p < 0.01 vs. 4 μM AZD3759). Furthermore, we suspected that the inhibitory effect of AZD3759 on the growth of glioma cells was associated with the inhibition of JAK signaling. As shown in Figure 2, in both C6 cells and U87 cells, p-JAK1/JAK1, p-JAK2/JAK2, p-STAT3/STAT3, and p-STAT5/STAT5 were significantly downregulated by the introduction of 2 μM and 4 μM AZD3759 (*p < 0.05 vs. control, **p < 0.01 vs. control). However, compared to the 4 μM AZD3759 group, the 4 μM osimertinib group had a significantly higher expression of p-JAK1/JAK1, p-JAK2/JAK2, p-STAT3/STAT3, and p-STAT5/STAT5 (##p < 0.01 vs. 4 μM AZD3759). These data indicated that AZD3759 had a superior inhibitory effect on the JAK/STAT signal pathway than osimertinib.

### Luciferase activity was positively correlated with the number of C6-LUC cells

**Figure 3.**
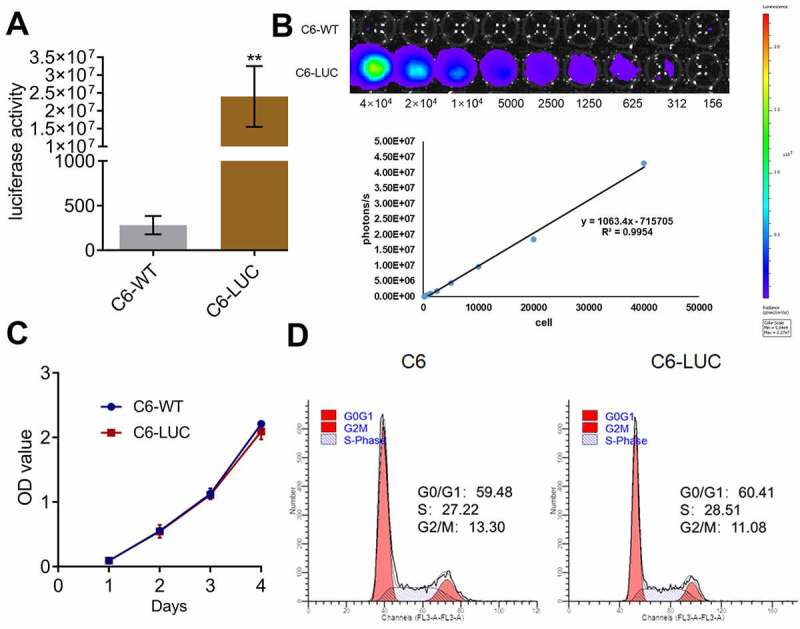
Luciferase activity is positively related to the number of C6-LUC cells. a. The luciferin activity in C6-WT and C6-LUC cells determined using the IVIS spectrum system (**p < 0.01 vs. C6-WT). b. The photons of the different number of C6-LUC cells detected by the IVIS spectrum system. c. The OD value of C6-WT and C6-LUC cells measured using CCK-8 assay. d. The cell cycle of C6-WT and C6-LUC cells determined using flow cytometry assay

We further explored the inhibitory effects of AZD3759 on the growth of glioma cells by monitoring the activity of luciferin in C6-LUC-implanted mice. To confirm whether the activity of luciferin could be used as an indicator for the proliferation of C6-LUC cells in *in vivo* experiments, the correlation between the activity of luciferin and the number of C6-LUC cells was determined. Only C6 murine glioma cells were used in subsequent *in vivo* experiments. Firstly, as shown in Figure 3a, compared to C6-wild-type (WT) cells, C6-LUC cells had a significantly elevated luciferin activity (**p < 0.01 vs. C6-WT). A linear correlation between photons and the number of cells was observed (Figure 3b), with an R^2^ value of 0.9954. In addition, no significant difference was observed in the OD values detected by the CCK-8 assay and in the G0/G1 cell fraction detected by flow cytometry between the C6-WT and C6-LUC cells, indicating that the proliferation and cell cycle of C6 cells were not affected by the transfection of luciferase.

### AZD3759 inhibited the growth of C6 cells in xenograft mice

**Figure 4.**
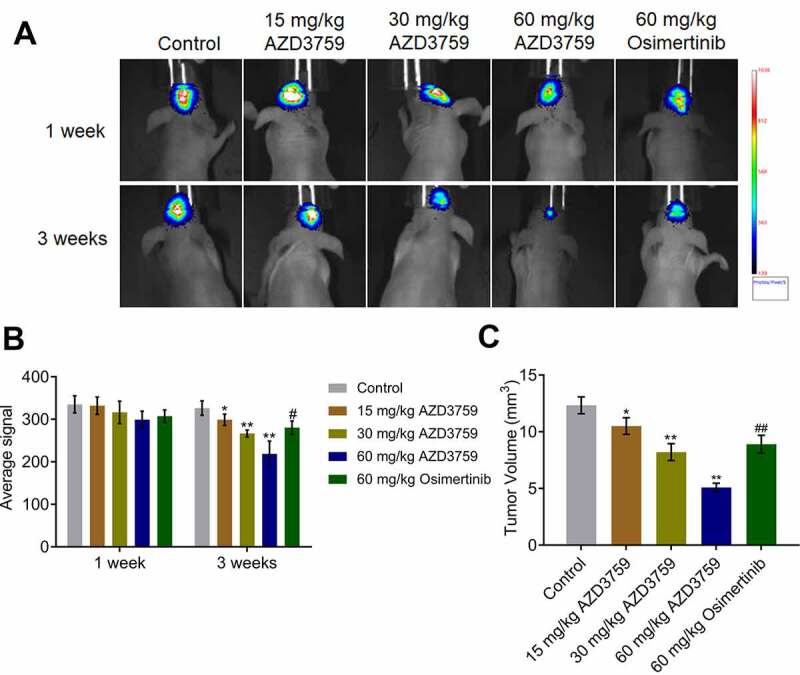
AZD3759 suppresses the growth of C6 cells in xenograft mice. a. Live images of animals of each group taken 1 week and 3 weeks after treatment. b. Average signals quantified using the IVIS spectrum system (*p < 0.05 vs. control, **p < 0.01 vs. control, #p < 0.05 vs. 60 mg/kg AZD3759). c. Tumor volumes calculated at the end of the experiments (*p < 0.05 vs. control, **p < 0.01 vs. control, ##p < 0.01 vs. 60 mg/kg AZD3759)

After confirmation of the positive correlation between the activity of luciferin and the number of C6-LUC cells, C6-LUC cells were implanted into the brain of mice to establish a xenograft animal model. As shown in Figure 4a and B, no significant difference in the average luciferin signal was observed in any of the groups 1 week after treatment with AZD3759 and osimertinib. However, 3 weeks after treatment with AZD3759 and osimertinib, the average luciferin signal was significantly reduced in the brain after administration of 15, 30, and 60 mg/kg AZD3759 compared to that after control treatment (*p < 0.05 vs. control, **p < . 0.01 vs. control). Compared to the 60 mg/kg AZD3759 group, the 60 mg/kg osimertinib group had a significantly higher signal (#p < 0.05 vs. 60 mg/kg AZD3759). In addition, at the end of the experiment, compared to the control group, the 15, 30, and 60 mg/kg AZD3759 groups had a dramatically smaller tumor volume in the brain (Figure 4c) (*p < 0.05 vs. control, **p < 0.01 vs. control). Compared to the 60 mg/kg AZD3759 group, the 60 mg/kg osimertinib group had a significantly larger tumor volume (##p < 0.01 vs. 60 mg/kg AZD3759). These data revealed that AZD3759 had superior inhibitory effect on tumor growth than osimertinib.

### AZD3759 suppressed the EGFR and JAK/STAT signaling pathways in xenograft C6 tissues

**Figure 5.**
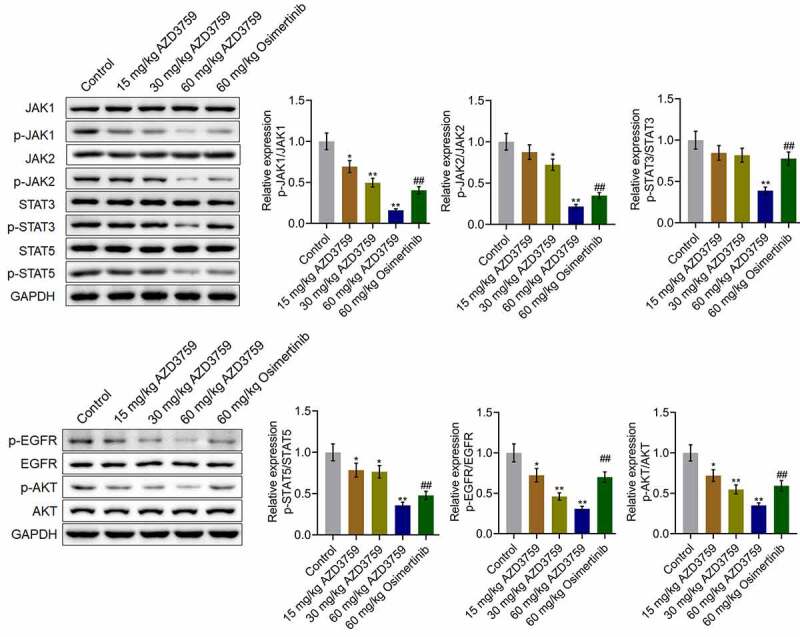
AZD3759 suppresses the EGFR and JAK/STAT signaling pathways in xenograft C6 tissues. The expression levels of JAK1, p-JAK1, JAK2, p-JAK2, STAT3, p-STAT3, STAT5, p-STAT5, EGFR, p-EGFR, AKT, and p-AKT in tumor tissues detected using western blot assay (*p < 0.05 vs. control, **p < 0.01 vs. control, ##p < 0.01 vs. 60 mg/kg AZD3759)

We further verified the inhibitory effect of AZD3759 on the EGFR and JAK/STAT signaling pathways in tumor tissues. As shown in Figure 5, p-EGFR/EGFR and p-AKT/AKT were dramatically downregulated by treatment with AZD3759 in a dose-dependent manner compared to those by control treatment (*p < 0.05 vs. control, **p < 0.01 vs. control). Compared to the 60 mg/kg AZD3759 group, the 60 mg/kg osimertinib group had higher expression levels of p-EGFR/EGFR and p-AKT/AKT (##p < 0.01 vs. 60 mg/kg AZD3759). The expression levels of p-JAK1/JAK1, p-JAK2/JAK2, p-STAT3/STAT3, and p-STAT5/STAT5 were significantly suppressed by treatment of AZD3759 in a dose-dependent manner compared to those by control treatment (*p < 0.05 vs. control, **p < 0.01 vs. control). In addition, compared to the 60 mg/kg AZD3759 group, the 60 mg/kg osimertinib group had a significant activation of the JAK/STAT signaling pathway (##p < 0.01 vs. 60 mg/kg AZD3759).

## Discussion

Osimertinib is an anilinopyrimidine compound with an irreversible and highly selective inhibitory effect on the T790M mutant EGFR [22]. During the determination of the recombinant enzyme of the EGFR gene, compared to the naïve EGFR gene, a 200-fold antagonist efficacy against the L858R/T790M EGFR mutant gene was observed, which verified the high selectivity of osimertinib against EGFR mutant genes [23]. In an *in vitro* model evaluating the specificity of targeting EGFR mutant genes, a wide therapeutic window of osimertinib was observed against the T790M mutant EGFR [24]. AZD3759 is an EGFR-TKI specially designed for the treatment of patients with brain metastatic NSCLC due to its ability to penetrate the BBB. Yang [14] reported the results of phase I clinical trials on AZD3759 in 2015, which showed high permeability through the BBB (29.5 × 10^−6^ cm/s) in patients with brain metastatic NSCLC. In addition, EGFR-mutant brain metastatic animal experiments revealed a significant decrease in tumor volume in AZD3759-treated mice [14]. In the present study, we suspected that osimertinib and AZD3759 might be effective for the treatment of glioma as both agents can penetrate the BBB. *In vitro* experiments indicated that in both human and murine glioma cells, proliferation was significantly inhibited and apoptosis was significantly induced by the introduction of AZD3759 in a dose-dependent manner, which was consistent with reports on the effects of AZD3759 against hepatoma cells [25] and NSCLC cells [14]. The anti-tumor efficacy of osimertinib and AZD3759 was further compared after incubating the glioma cells at the same concentration. We found superior efficacy in the AZD3759 group. However, the effective *in vitro* concentration of AZD3759 was in the μM range, which would limit the clinical applications of AZD3759 in the clinic. Combination therapy with chemotherapeutic drugs may be a promising strategy in the future. In addition, the correlation between the *in vitro* effective concentration and the achievable plasma/brain concentrations in animals or patients should be further investigated to explore the pharmacological properties of AZD3759 against glioma.

Luciferase is widely used to investigate the *in vivo* tracking of glioma cells [26,27]. No significant species differences were observed in the *in vitro* experiments. The *in vivo* experiment was conducted only on murine glioma cells by transplanting C6-LUC cells in mice. We first checked the correlation between the activity of luciferin and the number of C6-LUC cells to confirm that the activity of luciferin represents the *in vivo* growth of C6 cells, which was based on methods described in a previous study [28]. We found a dose-dependent inhibitory effect against the *in vivo* growth of C6 cells with AZD3759, which was consistent with the results observed in the *in vitro* assays. In addition, the superior anti-tumor efficacy of AZD3759 than that of osimertinib was verified in a xenograft animal model. Considering the BBB permeability ability of both osimertinib and AZD3759, we suspected that a specific regulatory mechanism might be involved in the anti-tumor efficacy of AZD3759 compared to that of osimertinib.

JAK is a member of the non-receptor tyrosine kinase and is subdivided into three phenotypes (JAK1, JAK2, and JAK3) as well as TYK2, which can activate STAT proteins on stimulation of multiple cytokines and growth factors [29–31]. The JAK/STAT pathway is strictly regulated in normal cells. However, in tumor cells, the JAK/STAT pathway can be persistently activated by the upstream pathway of JAK [32]. Activation of the JAK/STAT pathway has been widely reported in gliomas and has been shown to be involved in the development and progression of gliomas [33,34]. In the present study, we found that the inhibitory effect of AZD3759 on the JAK/STAT pathway was dose dependent in both glioma cells and tumor tissues. Compared to inhibitory effect of AZD3759 on the JAK/STAT pathway, that of osimertinib was relatively mild, which might be responsible for the uncompetitive anti-tumor properties observed in glioma cells and xenograft animal models. However, in our future work, this conclusion will be further verified by introducing osimertinib and AZD3759 into JAK-overexpressing glioma cells.

## Conclusion

In conclusion, AZD3759 may inhibit the progression of glioma through a synergistic blockade of the EGFR and JAK/STAT signaling pathways.

## Highlights

AZD3759 inhibits the progression of glioma.

AZD3759 suppresses the JAK and EGFR pathways.

AZD3759 has a superior antitumor effect against glioma than osimertinib.
